# Streptococcal Toxic Shock Syndrome Caused by Group G *Streptococcus*, United Kingdom

**DOI:** 10.3201/eid2301.161009

**Published:** 2017-01

**Authors:** Melissa Baxter, Marina Morgan

**Affiliations:** Royal Devon and Exeter National Health Service Foundation Trust, Devon, United Kingdom

**Keywords:** streptococcal toxic shock syndrome, STSS, streptococcus, bacteria, bacterial infections, group G streptococcus, intravenous immunoglobulin, IVIg, streptococci

## Abstract

We describe successful management of 3 patients with streptococcal toxic shock syndrome (STSS) attributable to group G *Streptococcus* infection. This small series supports recognition of group G *Streptococcus* in the etiology of STSS. We propose intravenous immunoglobulin be used in treatment as it is for STSS caused by group A *Streptococcus*.

Streptococcal toxic shock syndrome (STSS) was defined in 1993 as the isolation of group A *Streptococcus* (GAS) with parameters indicative of multiorgan dysfunction (*1*). STSS remains associated with high mortality rates, even with adequate antimicrobial treatment (*2*). Recently, 12 invasive infections with β-hemolytic streptococci of Lancefield group G also fulfilling the criteria of STSS have been reported (mortality rate 83%) (*3*–*12*). The benefit of adjunctive intravenous immunoglobulin (IVIg) in the treatment of STSS is difficult to prove because the limited number of cases precludes results of sufficient statistical power. Observational studies (*2*,*14*) and 1 small, early terminated, randomized control trial (*15*) have demonstrated improved survival among patients with GAS STSS treated with IVIg. We describe 3 patients treated in an 880-bed teaching hospital during 2009–2014; all 3 patients had illness meeting the case definition of STSS that were caused by invasive group G *Streptococcus* (GGS) infection. Successful treatment included IVIg, providing further support for the use of IVIg in treating STSS.

## The Patients

In 2009, a 46-year-old woman (patient 1) with multiple sclerosis and lower limb lymphedema was admitted to the hospital with a 1-week history of fever, vomiting, rigors, sore throat, and erythema of the right leg with a healing cut on the foot. Patchy cellulitis extended from the foot to groin and was associated with elevated body temperature (39.4°C). Her creatine kinase level was high at 3,951 IU/L, and she had an elevated C-reactive protein (CRP) level of 396 mg/L. Her illness was initially managed as severe cellulitis. She received intravenous fluids, clindamycin (1.2 g every 6 h), and flucloxacillin (1 g every 6 h). However, her condition rapidly deteriorated. She had onset of hypotension (blood pressure 77/24 mm Hg) refractory to 8 L fluid resuscitation, prompting transfer to the intensive therapy unit (ITU) for cardiovascular support. Computed tomography scan results were compatible with adult respiratory distress syndrome. Shock and spreading cellulitis (with patches appearing on the contralateral leg) suggested streptococcal myositis or multifocal necrotizing fasciitis. Flucloxacillin was changed to intravenous daptomycin (4 mg/kg daily), and meropenem (1 g every 8 h) and 2 g/kg polyclonal intravenous immunoglobulin (IVIg) (Privigen; CSL Behring, West Sussex, UK) were administered. Her condition continued to deteriorate. Erythema spread beyond skin markings, her CRP plateaued at levels >300 mg/L, and her serum IgG level was 4.6 mg/L, prompting administration of a second dose of IVIg 2 g/kg on day 3. Surgical exploration released 30 mL of fluid and revealed macroscopically normal fascia and muscle. Histologic evaluation revealed severely necrotic soft tissue. GGS was isolated from all operative specimens. A transthoracic echocardiogram showed no vegetations. Three weeks after admission, the patient was discharged on prophylactic penicillin and remains well.

In 2011, a 63-year-old previously healthy man (patient 2) was admitted to the hospital with a 5-day history of confusion, headache, and bilateral swollen painful knees. Among purpuric lesions on the left lower leg was a small healing wound. Multiorgan failure and septic shock necessitated transfer to the ITU for inotropic support and hemofiltration. Blood tests revealed a reduced platelet count of 54 × 10^9^/L and elevated levels of creatinine at 210 µmol/L, serum lactate at 10.2 mmol/L, CRP at 353 mg/L, and creatine kinase at 2,080 IU/L. Culture of joint fluid from each knee revealed GGS. The patient was treated empirically with intravenous imipenem (500 mg every 12 h), clindamycin (1.8 g every 6 h), vancomycin (1 g stat), and 2 g/kg polyclonal IVIg (Privigen). Despite dramatic clinical improvement, a static CRP level and worsening pain in other joints necessitated repeated arthroscopic washouts of his knees, wrists, hips, and 1 shoulder. Although gram-positive cocci were observed on microscopic evaluation of the knee and shoulder fluids, all aspirates and blood cultures were sterile. A transesophageal echocardiogram found no evidence of endocarditis; however, a repeat transesophageal echocardiogram 1 month after admission revealed a vegetation, necessitating aortic valve replacement. The patient was discharged after 13 weeks and remains well.

In 2014, a 66-year-old man (patient 3) with a history of diabetes mellitus was admitted to the hospital in septic shock. A total knee replacement 6 months earlier had resulted in chronic leg lymphedema. Noteworthy blood test results included a high serum lactate level of 4 mmol/L, an elevated creatinine level of 318 µmol/L, and a high alanine aminotransferase level of 210 IU/L. The patient had a blood pressure of 40/20 mm Hg, rapidly spreading erythema from the knee to the abdomen, and suffused conjunctivae. His illness was managed clinically as toxic shock syndrome. He received inotropic support, mechanical ventilation, hemofiltration, and intravenous linezolid (600 mg every 12 h), intravenous clindamycin (1.2 g every 6 h), and 2 g/kg polyclonal IVIg (Privigen). Arthroscopic washout of the prosthetic knee revealed copious pus that yielded GGS on culture. Although cardiovascular function stabilized, blood parameters continued to deteriorate. On day 3, following open debridement of the knee, the patient began to improve, and his CRP level remained elevated at 280 mg/L. Antimicrobial drugs were changed to intravenous daptomycin (7 mg/kg once daily) to maximize antibiofilm activity (reference *16* in Technical Appendix). After a negative echocardiography, the patient was discharged home on day 30, completing a 6-week course of daptomycin as an outpatient, and successfully retained the knee prosthesis. He remains well on life-long penicillin prophylaxis.

## Conclusions

We describe 3 patients with group G STSS. Isolates were identified by laboratory serotyping of the group G carbohydrate surface antigen. All 3 patients had a favorable outcome after aggressive therapy with a combination of antiexotoxin antibiotics, IVIg, and surgical intervention. 

The emergence of GGS causing skin and soft tissue infections (reference *17* in Technical Appendix) might reflect improved detection, increased virulence, or a growing population of immunocompromised hosts. Similar to GAS, GGS shares multiple virulence factors, including streptokinase, fibronectin, IgG binding proteins, streptolysin O, C5a peptidase (reference *18* in Technical Appendix), and antiphagocytic M proteins, of which e*mm* types *stg10* and *stg2078* of GGS are significantly associated with invasive disease (reference *19* in Technical Appendix). Given clinical presentations of STSS by group G and group C *Streptococcus* are indistinguishable from group A STSS, the underlying mechanisms are probably related. However, unlike group A STSS, with group G STSS, underlying co-morbidities predominate, including cardiopulmonary disease, diabetes mellitus, malignancy, or hepatic failure (*6*). 

IVIg has superantigen neutralizing activity (reference *20* in Technical Appendix) and reduces mortality rates in group A STSS (*2*,*14*,*15*) A recent review of IVIg use for severe sepsis and septic shock yielded little evidence of benefit (reference *21* in online Technical Appendix). However, in that review, there was no discrimination between superantigen, exotoxin-driven, gram-positive sepsis and primarily endotoxin-driven, gram-negative sepsis, and very few cases of gram-positive sepsis were included (reference *21* in Technical Appendix). 

Twelve cases of group G STSS have been previously documented (*3*–*12*); 10 of those patients died (mortality rate 83%). With the addition of the 3 cases we reported, group G STSS has an overall reported mortality rate of 66%. The use of IVIg was described in the cases of 4 patients, 3 of whom died (*5*,*7*,*11*,*12*), including 1 who received low-dose IVIg (400 mg/kg) (*12*); for the other patients, the IVIg dosage was unspecified. Although our case series is small, it describes long-term survival in 3 patients with group G STSS. We propose that the definition of STSS extends beyond that of a condition exclusive to GAS infection and recommend that polyclonal immunoglobulin be considered as a potentially lifesaving adjunctive therapy.

Technical Appendix References for citations 16–21.
