# Supplementary material for: Streptococcal Toxic Shock Syndrome Caused by Group G Streptococcus, United Kingdom
Source: Emerg Infect Dis. 2017 Jan;23(1):127–9. doi: 10.3201/eid2301.161009 (PMC5176243; doi:10.3201/eid2301.161009)
Supplement: Technical Appendix — References for citations 16–21. [file 16-1009-Techapp-s1.pdf]

Article DOI: http://dx.doi.org/10.3201/eid2301.161009

Streptococcal Toxic Shock Syndrome Caused by Group G *Streptococcus*, United Kingdom

Technical Appendix

Additional References

16. Leite B, Gomes F, Teixeira P, Souza C, Pizzolitto E, Oliveira R. In vitro activity of daptomycin, linezolid and rifampicin on *Staphylococcus epidermidis* biofilms. Curr Microbiol. 2011;63:313–7. PubMed http://dx.doi.org/10.1007/s00284-011-9980-7

17. Oppegaard O, Mylvaganam H, Kittang BR. Beta-haemolytic group A, C and G streptococcal infections in Western Norway: a 15-year retrospective survey. Clin Microbiol Infect. 2015;21:171–8. PubMed http://dx.doi.org/10.1016/j.cmi.2014.08.019

18. Humar D, Datta V, Bast DJ, Beall B, De Azavedo JC, Nizet V. Streptolysin S and necrotising infections produced by group G streptococcus. Lancet. 2002;359:124–9. PubMed http://dx.doi.org/10.1016/S0140-6736(02)07371-3

19. Pinho MD, Melo-Cristino J, Ramirez M. Clonal relationships between invasive and noninvasive Lancefield group C and G streptococci and *emm*-specific differences in invasiveness. J Clin Microbiol. 2006;44:841–6. PubMed http://dx.doi.org/10.1128/JCM.44.3.841-846.2006

20. Norrby-Teglund A, Kaul R, Low DE, McGeer A, Andersson J, Andersson U, et al. Evidence for the presence of streptococcal-superantigen-neutralizing antibodies in normal polyspecific immunoglobulin G. Infect Immun. 1996;64:5395–8. PubMed

21. Alejandria MM, Lansang MAD, Dans LF, Mantaring JB III. Intravenous immunoglobulin for treating sepsis, severe sepsis and septic shock. Cochrane Database Syst Rev. 2013;9:CD001090. PubMed http://dx.doi.org/10.1002/14651858.CD001090.pub2
